# Cannabinoids and Pain: New Insights From Old Molecules

**DOI:** 10.3389/fphar.2018.01259

**Published:** 2018-11-13

**Authors:** Sonja Vučković, Dragana Srebro, Katarina Savić Vujović, Čedomir Vučetić, Milica Prostran

**Affiliations:** ^1^Department of Pharmacology, Clinical Pharmacology and Toxicology, Faculty of Medicine, University of Belgrade, Belgrade, Serbia; ^2^Clinic of Orthopaedic Surgery and Traumatology, Clinical Center of Serbia, Belgrade, Serbia; ^3^Faculty of Medicine, University of Belgrade, Belgrade, Serbia

**Keywords:** cannabis/cannabinoids, pain, pharmacodynamics, pharmacokinetics, efficacy, safety, animals, humans

## Abstract

Cannabis has been used for medicinal purposes for thousands of years. The prohibition of cannabis in the middle of the 20th century has arrested cannabis research. In recent years there is a growing debate about the use of cannabis for medical purposes. The term ‘medical cannabis’ refers to physician-recommended use of the cannabis plant and its components, called cannabinoids, to treat disease or improve symptoms. Chronic pain is the most commonly cited reason for using medical cannabis. Cannabinoids act via cannabinoid receptors, but they also affect the activities of many other receptors, ion channels and enzymes. Preclinical studies in animals using both pharmacological and genetic approaches have increased our understanding of the mechanisms of cannabinoid-induced analgesia and provided therapeutical strategies for treating pain in humans. The mechanisms of the analgesic effect of cannabinoids include inhibition of the release of neurotransmitters and neuropeptides from presynaptic nerve endings, modulation of postsynaptic neuron excitability, activation of descending inhibitory pain pathways, and reduction of neural inflammation. Recent meta-analyses of clinical trials that have examined the use of medical cannabis in chronic pain present a moderate amount of evidence that cannabis/cannabinoids exhibit analgesic activity, especially in neuropathic pain. The main limitations of these studies are short treatment duration, small numbers of patients, heterogeneous patient populations, examination of different cannabinoids, different doses, the use of different efficacy endpoints, as well as modest observable effects. Adverse effects in the short-term medical use of cannabis are generally mild to moderate, well tolerated and transient. However, there are scant data regarding the long-term safety of medical cannabis use. Larger well-designed studies of longer duration are mandatory to determine the long-term efficacy and long-term safety of cannabis/cannabinoids and to provide definitive answers to physicians and patients regarding the risk and benefits of its use in the treatment of pain. In conclusion, the evidence from current research supports the use of medical cannabis in the treatment of chronic pain in adults. Careful follow-up and monitoring of patients using cannabis/cannabinoids are mandatory.

## Introduction

Pain is one of the most common symptoms of disease. Acute pain is usually successfully managed with non-steroidal anti-inflammatory drugs (NSAIDs) and/or opioids (Vučković S. et al., 2006; Vučković S.M. et al., 2006; Vučković et al., 2009, Vučković et al., 2016), but chronic pain is often difficult to treat and can be very disabling (Gatchel et al., 2014). An adjuvant is a drug that is not primarily intended to be an analgesic but can be used to reduce pain either alone or in combination with other pain medications (Bair and Sanderson, 2011). Some of these drugs have been known for some time, but their acceptance has waxed and waned over time (Vučković et al., 2015; Srebro et al., 2016; Tomić et al., 2018). However, new approaches to targeting the pain pathway have been developed and adjuvant analgesics continue to attract both scientific and medical interest as constituents of a multimodal approach to pain management (Yaksh et al., 2015). The role of cannabis plant and its components, called cannabinoids, as adjuvant analgesics in the treatment of chronic pain, has been the subject of longstanding controversy (NASEM, 2017).

Flowering plants within the genus Cannabis (also known as marijuana) in the family Cannabaceae have been cultivated for thousands of years in many parts of the world for spiritual, recreational and medicinal purposes. Preparations of the cannabis plant, which are taken by smoking or oral ingestion, have been observed to produce analgesic, anti-anxiety, anti-spasmodic, muscle relaxant, anti-inflammatory and anticonvulsant effects (Andre et al., 2016). However, the prohibition of cannabis cultivation, supply and possession from the middle of the 20th century (due to its psychoactivity and potential for producing dependence), has impeded cannabis research (ElSohly et al., 2017). In recent years there is a growing debate about cannabis use for medical purposes. In many countries cannabis use for medical reasons is legal and some countries have also decriminalized or legalized the recreational use of cannabis.

The term medical cannabis is used to refer to the physician-recommended use of cannabis and its constituents, cannabinoids, to treat disease or improve symptoms (Rahn and Hohmann, 2009). The use of cannabis and cannabinoids may be limited by its psychotropic side effects (e.g., euphoria, anxiety, paranoia) or other central nervous system (CNS)-related undesired effects (cognitive impairment, depression of motor activity, addiction), which occur because of activation of cannabinoid CB1 receptors in the CNS (Volkow et al., 2014). As interest in the use of cannabinoids as adjunctive therapy for pain management has increased in the last decades (Hill et al., 2017), there has been a continuing need for an increase in cannabis research and bridging the knowledge gap about cannabis and its use in pain treatment. Therefore, research on cannabis and cannabinoids has increased dramatically in recent years. However, there are several obstacles that need to be overcome, such as the regulations and policies that restrict access to the cannabis products, funding limitations, and numerous methodological challenges (drug delivery, the placebo issue, etc.) (NASEM, 2017). This research is expected to explain and update the mechanisms of analgesic action of cannabis and its constituents, and to provide answers to questions about the safety of medicinal cannabis and its potential indications in the treatment of pain. Healthcare providers in all parts of the world must keep up to date with recent findings in order to provide valid information regarding the benefits, risks, and responsible medical use to patients in pain (Wilsey et al., 2016).

This article is a narrative review of the published preclinical and clinical research of the pharmacodynamics, pharmacokinetics, efficacy, safety and tolerability of cannabis/cannabinoids in the treatment of pain.

## Materials and Methods

In March 2018 we searched the MEDLINE database via PubMed (United States National Library of Medicine) for articles published up to March 1st, 2018 for the key words: ‘cannabis’ or ‘cannabinoids’ and ‘pain’ (in title/abstract). This was followed by filter species (humans/other animals) and language (English) selection. The abstracts of the 1270 citations extracted were screened for relevance by two reviewers (SV and DS). Discrepancies were resolved by discussion. The literature relevant to pharmacodynamic, pharmacokinetics, efficacy and safety of cannabis/cannabinoids in pain treatment was included. Both preclinical *in vitro* and *in vivo* data and clinical studies were included. Data on cannabis use among children, adolescents and pregnant women were excluded. We also examined the reference lists of reviewed articles.

## Pharmacodynamics: Cannabis and Cannabinoids Act on Multiple Pain Targets

For many years it was assumed that the chemical components of the cannabis plant, cannabinoids, produce analgesia by activating specific receptors throughout the body, in particular CB1, which are found predominantly in the CNS, and CB2, found predominantly in cells involved with immune function (Rahn and Hohmann, 2009). However, recently this picture has become much more complicated, as it has been recognized that cannabinoids, both plant-derived and endogenous, act simultaneously on multiple pain targets (Ross, 2003; Horvath et al., 2008; Pertwee et al., 2010; O’Sullivan, 2016; Morales et al., 2017) within the peripheral and CNS. Beside acting on cannabinoid CB1/CB2 receptors, they may reduce pain through interaction with the putative non-CB1/CB2 cannabinoid G protein-coupled receptor (GPCR) 55 (GPR55; Staton et al., 2008) or GPCR 18 (GPR18), also known as the *N*-arachidonoyl glycine (NAGly) receptor; Huang et al., 2001), and other well-known GPCRs, such as the opioid or serotonin (5-HT) receptors (Russo et al., 2005; Scavone et al., 2013). In addition, many studies have reported the ability of certain cannabinoids to modulate nuclear receptors (peroxisome proliferator-activated receptors (PPARs) (O’Sullivan, 2016), cys loop ligand-gated ion channels (Barann et al., 2002; Hejazi et al., 2006 Ahrens et al., 2009; Sigel et al., 2011; Xiong et al., 2011, 2012; Shi et al., 2012; Oz et al., 2014; Bakas et al., 2017) or transient receptor potential (TRP) channels (TRPV, TRPA, and TRPM subfamilies), (Pertwee et al., 2010; Lowin and Straub, 2015; Morales et al., 2017), among others. It has been shown that all these receptors represent potentially attractive targets for the therapeutic use of cannabinoids in the treatment of pain. Moreover, TRPV1 and CB1 or CB2 are colocalized at peripheral and/or central neurons (sensory neurons, dorsal root ganglia, spinal cord, brain neurons), which results in their intracellular crosstalk in situations where these receptors are involved simultaneously (Cristino et al., 2006; Anand et al., 2009). New data also demonstrate a variety of interactions between cannabinoid, opioid, and TRPV1 receptors in pain modulation (Zádor and Wollemann, 2015). All of these provide an opportunity for the development of new multiple target ligands and polypharmacological drugs with improved efficacy and devoid of side effects for the treatment of pain (Reddy and Zhang, 2013).

Several lines of evidence indicate that cannabinoids may contribute to pain relief through an anti-inflammatory action (Jesse Lo et al., 2005; Klein, 2005). In addition, non-cannabinoid constituents of the cannabis plant that belong to miscellaneous groups of natural products (terpenoids and flavonoids) may contribute to the analgesic, as well as the anti-inflammatory effects of cannabis (Andre et al., 2016; ElSohly et al., 2017).

Based on their origin, cannabinoids are classified into three categories: phytocannabinoids (plant-derived), endocannabinoids (present endogenously in human or animal tissues), and synthetic cannabinoids.

### Phytocannabinoids

There are about 100 different cannabinoids isolated from the cannabis plant (Andre et al., 2016). The main psychoactive compound is (-)-*trans*-Δ9-tetrahydrocannabinol (THC), which is produced mainly in the flowers and leaves of the plant. The THC content varies from 5% in marijuana to 80% in hashish oil. THC is an analog to the endogenous cannabinoid, anandamide (ananda is the Sanskrit word for bliss; arachidonoylethanolamide, AEA). It is responsible for most of the pharmacological actions of cannabis, including the psychoactive, analgesic, anti-inflammatory, anti-oxidant, antipruritic, bronchodilatory, anti-spasmodic, and muscle-relaxant activities (Rahn and Hohmann, 2009; Russo, 2011). THC acts as a partial agonist at cannabinoid receptors (CB1 and CB2) (Pertwee, 2008). A very high binding affinity of THC with the CB1 receptor appears to mediate its psychoactive properties (changes in mood or consciousness), memory processing, motor control, etc. It has been reported that a number of side effects of THC, including anxiety, impaired memory and immunosuppression, can be reversed by other constituents of the cannabis plant (cannabinoids, terpenoids, and flavonoids) (Russo and Guy, 2006; Russo, 2011; Andre et al., 2016).

The non-psychoactive analog of THC, cannabidiol (CBD), is another important cannabinoid found in the cannabis plant. It is thought to have significant analgesic, anti-inflammatory, anti-convulsant and anxiolytic activities without the psychoactive effect of THC (Costa et al., 2007). CBD has little binding affinity for either CB1 or CB2 receptors, but it is capable of antagonizing them in the presence of THC (Thomas et al., 2007). In fact, CBD behaves as a non-competitive negative allosteric modulator of CB1 receptor, and it reduces the efficacy and potency of THC and AEA (Laprairie et al., 2015). CBD also regulates the perception of pain by affecting the activity of a significant number of other targets, including non-cannabinoid GPCRs (e.g., 5-HT1A), ion channels (TRPV1, TRPA1 and TPRM8, GlyR), PPARs, while also inhibiting uptake of AEA and weakly inhibiting its hydrolysis by the enzyme fatty acid amide hydrolase (FAAH) (Russo et al., 2005; Staton et al., 2008; Ahrens et al., 2009; De Petrocellis et al., 2011; Burstein, 2015; Morales et al., 2017). It has been demonstrated that cannabidiol can act synergistically with THC and contribute to the analgesic effect of medicinal-based cannabis extract (Russo, 2011). At the same time, CBD displays an entourage effect (the mechanism by which non-psychoactive compounds present in cannabis modulate the overall effects of the plant), and is capable of improving tolerability and perhaps also the safety of THC by reducing the likelihood of psychoactive effects and antagonizing several other adverse effects of THC (sedation, tachycardia, and anxiety) (Russo and Guy, 2006; Abrams and Guzman, 2015). The differences in concentration of THC and CBD in the plant reflect the differences in the effects of different cannabis strains. Although CBD as a monotherapy in the treatment of pain has not been evaluated clinically, its anti-inflammatory (Ko et al., 2016) and anti-spasmodic benefits and good safety profile suggest that it could be an effective and safe analgesic (Wade et al., 2003).

Other phytocannabinoids that can contribute to the overall analgesic effects of medical cannabis are cannabichromene (CBC), cannabigerol (CBG), tetrahydrocannabivarin (THCV), and many others (Morales et al., 2017). Similarly to CBD, these compounds do not display significant affinities for cannabinoid receptors, but they have other modes of action. This is a new area of research that needs to be addressed (Piomelli et al., 2017).

### Endocannabinoid System

This system seems to regulate many functions in the body, including learning and memory, mood and anxiety, drug addiction, feeding behavior, perception, modulation of pain and cardiovascular functions. The endocannabinoid system consists of cannabinoid receptors, endogenous cannabinoids (endocannabinoids), transport proteins and enzymes that synthesize or degrade the endocannabinoids.

Cannabinoid CB1 and CB2 receptors are 7-transmembrane G-protein coupled receptors (GPCRs). They play an important role in peripheral, spinal, and supraspinal nociception, including ascendant and descendent pain pathways (Hill et al., 2017). The signal transduction pathway of CB1 and CB2 involves inhibition of adenylyl cyclase, decreased cAMP formation, as well as an increase in the activity of mitogen-activated protein kinases (MAPK) (Ibsen et al., 2017). New evidence is emerging that different ligands can differentially activate these pathways, suggesting biased signaling through the cannabinoid receptors CB1 and CB2 (Ibsen et al., 2017).

The CB1 receptor is distributed throughout the nervous system. It mediates psychoactivity, pain regulation, memory processing and motor control. CB1 is a presynaptic heteroreceptor that modulates neurotransmitter and neuropeptide release and inhibits synaptic transmission. Activation of CB1 results in the activation of inwardly rectifying potassium channels, which decrease presynaptic neuron firing, and in the inhibition of voltage-sensitive calcium channels that decrease neurotransmitter release (Morales et al., 2017). The CB1 receptor is strategically located in regions of the peripheral and CNS where pain signaling is intricately controlled, including the peripheral and central terminals of primary afferent neurons, the dorsal root ganglion (DRG), the dorsal horn of the spinal cord, the periaqueductal gray matter, the ventral posterolateral thalamus and cortical regions associated with central pain processing, including the anterior cingulate cortex, amygdala and prefrontal cortex (Hill et al., 2017). The principal endogenous ligand for the CB1 receptor is AEA. CB1 receptors are observed more often on the gamma-aminobutyric acid (GABA) inhibitory interneurons in the dorsal horn of the spinal cord, and weakly expressed in most excitatory neurons (Hill et al., 2017). CB1 receptors are also present in multiple immune cells such as macrophages, mast cells and epidermal keratinocytes.

The CB2 receptor is found predominantly at the periphery (in tissues and cells of the immune system, hematopoietic cells, bone, liver, peripheral nerve terminals, keratinocytes), but also in brain microglia (Abrams and Guzman, 2015). The receptors are responsible for the inhibition of cytokine/chemokine release and neutrophil and macrophage migration and they contribute to slowing down of chronic inflammatory processes and modulate chronic pain (Niu et al., 2017). Both CB2 and CB1 receptors on mast cells participate in the anti-inflammatory mechanism of action of cannabinoids (Facci et al., 1995; Small-Howard et al., 2005). Also, activation of CB2 receptors on keratinocytes stimulates the release of β-endorphin, which acts at μ opioid receptors on peripheral sensory neurons to inhibit nociception (Ibrahim et al., 2005). Under basal conditions, CB2 receptors are present at low levels in the brain, the spinal cord and DRG, but may be upregulated in microglia where they modulate neuroimmune interaction in inflammation and after peripheral nerve damage (Hsieh et al., 2011). CB2 receptor activation inhibits adenylyl cyclase activity and stimulates MAPK activity, but the effect on calcium or potassium conductance is controversial (Rahn and Hohmann, 2009; Atwood et al., 2012). Stimulation of CB2 receptors does not produce cannabis-like effects on the psyche and circulation. The principal endogenous ligand for the CB2 receptor is 2-arachidonoylglycerol (2-AG) (Kano, 2014).

Endocannabinoids are arachidonic acid derivatives. AEA and 2-AG are synthesized separately, they have local (autocrine and paracrine) effects and are rapidly removed by hydrolysis by fatty acid amide hydrolase (FAAH) and monoacylglycerol lipase (MAGL), respectively (Pacher et al., 2006; Starowicz and Przewlocka, 2012; Howard et al., 2013). Beside AEA, FAAH inhibition significantly elevates the levels of other fatty-acid amides such as oleoylethanolamide (OEA) and palmitoylethanolamide (PEA) in the CNS and peripheral tissues (Lambert et al., 2002). Endocannabinoids, similarly to THC, appear to activate cannabinoid receptors. AEA and 2-AG are a partial and full agonist of CB receptors, respectively (Kano, 2014). They work as a part of a negative feedback loop that regulates neurotransmitter and neuropeptide release and thereby modulate various CNS functions, including pain processing (Vaughan and Christie, 2005).

The AEA is a full agonist at TRPV1 (AEA referred to as an ‘endovanilloid’) that activates TRPV1 which results in desensitization (Ross, 2003; Horvath et al., 2008; Starowicz and Przewlocka, 2012). AEA also activates GR55 (Ryberg et al., 2007), directly inhibits 5-HT3A receptors (Barann et al., 2002) potentiates the function of glycine receptors (Hejazi et al., 2006), inhibits T-type voltage-gated calcium channels (Chemin et al., 2001) and activates PPARs (Rockwell and Kaminski, 2004; Sun et al., 2007; Romano and Lograno, 2012; O’Sullivan, 2016).

Endocannabinoids, which are produced in neural and non-neural cells in the physiological response to tissue injury or excessive nociceptive signaling, suppress inflammation, sensitization and pain (Piomelli and Sasso, 2014; Maccarrone et al., 2015). Inhibitors of FAAH lead to elevated AEA levels and are intended for therapeutic use (Hwang et al., 2010). *N*-acylethanolamines such as PEA and OEA do not belong to endocannabinoids as they do not bind to cannabinoid receptors; they exhibit anti-inflammatory action via PPARs, and also inhibit pain through TRPV1 receptors. They are of interest to the field of cannabinoid pain research as they elevate levels of AEA through substrate competition at FAAH (Lambert et al., 2002).

There is a constant active exchange of substrates and metabolites between endocannabinoid and eicosanoid pathways. The enzyme FAAH breaks down AEA to arachidonic acid and ethanolamine or, alternatively, AEA can be directly transformed by cyclooxygenase-2 (COX-2) into proalgesic prostaglandins. As such, AEA may contribute to the analgesic properties of COX-2 selective NSAIDs. It was established that the metabolite of paracetamol combines with arachidonic acid by the action of FAAH to produce an endocannabinoid, which is a potent agonist at the TRPV1 and a weak agonist at both CB1 and CB2 receptors and an inhibitor of AEA reuptake (Bertolini et al., 2006).

### Synthetic Cannabinoids

At present, there are two synthetic cannabinoids on the market, dronabinol and nabilone, which may be of benefit in the treatment of pain (Abrams and Guzman, 2015). In general, their use in pain treatment is off-label. Dronabinol is a generic name for the oral form of synthetic THC (Marinol^®^). It is approved for the treatment of chemotherapy-associated nausea and vomiting, and anorexia associated with human immunodeficiency virus infection. Nabilone, a generic name for the orally administered synthetic structural analog of THC (Cesamet^®^), is approved for the treatment of chemotherapy-associated nausea and vomiting. Their medical use is mostly limited by their psychoactive side effects, as well as their limited bioavailability (Huestis, 2007).

### Cannabis and Cannabis Extract

Cannabis delivered by way of inhalation (smoked or inhaled through vaporization), orally or oromucosally, produces a host of biological effects (Andre et al., 2016). Unfortunately, clinical trials conducted on cannabis are limited, and no drug agency has approved the use of cannabis as a treatment for any medical condition. Although there is no formal approval, cannabis is widely used for the treatment of pain. It is authorized by physicians where medical marijuana is legal (Health Canada, 2013).

Nabiximols, a generic name for the whole-plant extract with a 1:1 ratio of THC:CBD (2.7 THC + 2.5 CBD per 100 μL), an oromucosal spray (Sativex^®^) is approved as an adjuvant treatment for symptomatic relief of spasticity in adult patients with multiple sclerosis (MS) who have not responded well to other therapy and who have demonstrated a significant improvement during an initial trial of Sativex^®^ therapy. In addition, Sativex^®^ is approved in Canada (under the Notice of Compliance with Conditions) as an adjuvant treatment for symptomatic relief of neuropathic pain in adults with MS, and as an adjuvant analgesic in adult patients with advanced cancer who suffer from moderate to severe pain that is resistant to strong opioids (Health Canada, 2013). An approval under the Notice of Compliance with Conditions means that a product shows potential benefit, possesses high quality and an acceptable safety profile based on a benefit-risk evaluation (Portenoy et al., 2012). Nabiximols is also approved in the United Kingdom and some European countries (e.g., Spain). The United States Food and Drug Administration (FDA) has not yet approved nabiximols as a treatment for any medical condition. Currently it is under investigation by the FDA under the Investigational New Drug Application (IND) for the treatment of cancer pain. Beside THC and CBD, nabiximols also contains other cannabinoids, terpenoids, and flavonoids.

## Pharmacokinetics of Cannabis/Cannabinoids

Cannabis is mostly inhaled by smoking and to a lesser extent by vaporization. The pharmacokinetics of inhaled and oral cannabis differ significantly (Agurell et al., 1986; Huestis, 2007). Taken by mouth, THC is metabolized in the liver to 11-hydroxy-THC, a potent psychoactive metabolite. By inhalation, cannabis (THC) avoids the first passage metabolism in the liver, and the effect of inhaled cannabis is proportionate to the plasma levels of THC. The pharmacokinetic profile of the inhaled cannabis is similar to THC given by the intravenous route (Agurell et al., 1986). The pharmacokinetic profile of CBD is very similar to THC given by the same route of administration.

When inhaled, cannabinoids are rapidly absorbed into the bloodstream. The advantages of inhaled over oral cannabis are the fast onset of action (requiring minutes instead of hours), and rapid attainment of peak effect (in 1 h vs. several hours), which is maintained at a steady level for 3–5 h (vs. the variable effect, observed after oral administration, which lasts from 8 to more than 20 h) and less generation of the psychoactive metabolite (Agurell et al., 1986). The analgesic effect is experienced shortly after the first breath and can be maximized by self-titration (patients adjust cannabis dosage themselves). However, self-titration of oral cannabis is not recommended due to the unpredictable appearance of side effects. The main disadvantage of smoking cannabis is inhalation of combustion byproducts with possible adverse effects in the respiratory tract (Volkow et al., 2014; NASEM, 2017). Therefore, vaporization is considered a better alternative for the inhalation of cannabis. About 25–27% of the available THC becomes available to the systemic circulation after smoking (Carter et al., 2004; Zuurman et al., 2009). The bioavailability of inhaled THC varies considerably, probably due to differences in inhalation techniques and source of the cannabis product (Agurell et al., 1986; Huestis, 2007).

Dronabinol, nabilone, and nabiximols are currently available oral pharmaceutical preparations of cannabinoids with standardized concentrations or doses. The main limitation associated with the administration of oral cannabinoids is their poor pharmacokinetic profile characterized by slow, unpredictable and highly variable absorption, late onset of action, extended duration due to psychoactive metabolites and unpredictable psychotropic effects (Ohlsson et al., 1980; Huestis, 2007; Issa et al., 2014). Oral THC (extract, synthetic or cannabis-derived) bioavailability was reported to be 6–20% only (Wall et al., 1983; Agurell et al., 1986). Further efforts are aimed at improving the bioavailability of oral cannabinoids (Smith, 2015).

Tetrahydrocannabinol is characterized by high binding to plasma protein (95–99%) so that the initial volume of distribution of THC is equivalent to the plasma volume (Grotenhermen, 2003). However, the distribution changes over time, with the steady state volume of distribution being about 3.5 L per kg of body weight. This is due to the high lipophilicity of THC, with high binding to fat tissue. THC crosses the placental barrier and small amounts also cross into breast milk (Grotenhermen, 2003).

Tetrahydrocannabinol is metabolized by cytochrome P450 enzymes CYP 2C9, 2C19 and 3A4, (Huestis, 2007; Rong et al., 2018), and drugs that inhibit these enzymes (e.g., proton pump inhibitors, HIV protease inhibitors, macrolides, azole antifungals, calcium antagonists and some anti-depressants) can increase the bioavailability of THC. Conversely, drugs that induce hepatic enzymes responsible for THC metabolism (e.g., phenobarbital, phenytoin, troglitazone, and St John’s wort) will lower its bioavailability (Rong et al., 2018).

In chronic-pain patients on opioid therapy, vaporized cannabis increases the analgesic effects of opioids without affecting significantly the plasma opioid levels (Abrams et al., 2011) suggesting that the effects are probably due to pharmacodynamic rather than pharmacokinetic interactions.

## Cannabinoids in Animal Models of Pain

Behavioral studies have shown that synthetic or plant-derived cannabinoid receptor agonists or endogenous cannabinoid ligands are effective in different animal models of acute pain (Dhopeshwarkar and Mackie, 2014). However, data obtained in humans, including volunteers with experimental pain and clinical trial patients, suggest that cannabinoids may be more effective for chronic rather than acute pain conditions (Kraft et al., 2008). Also, a number of targets identified in animal studies have not been confirmed in clinical trials. These include the absence of apparent clinical activity in clinical trials with CB2 agonists (Roche and Finn, 2010; Ostenfeld et al., 2011; Atwood et al., 2012; Pereira et al., 2013; Dhopeshwarkar and Mackie, 2014). In addition, FAAH inhibitors, although providing promising data in animal studies, did not demonstrate a significant efficacy against chronic pain in humans (Huggins et al., 2012; Woodhams et al., 2017). These discrepancies may be explained by species differences, differences in methodology and outcomes measured in the studies, as well as lack of selectivity of the ligands used (Dhopeshwarkar and Mackie, 2014). On the other hand, the outcome of a clinical trial of pain depends on the type of pain, trial design, target patient population, and several other factors (Gewandter et al., 2014). The effect of THC and other cannabinoids acting at CB1 receptors on motor activity in animals may easily be misinterpreted as pain-suppressing behavior (Meng et al., 1998). In humans, multiple emotional and cognitive factors influence the perception and experience of pain and this result in high inter-individual variability. However, pain in animals is mainly measured as a behavioral response to noxious stimuli, so that results obtained from animal studies are often more consistent. Also, volunteers with experimental pain respond more uniformly than patients with pathological pain, and pain pathways in healthy volunteers differ from those in patients (Olesen et al., 2012).

Due to CB1 receptor activation, the cannabinoid antinociception in animals may be accompanied by CNS side effects (e.g., hypoactivity, hypothermia and catalepsy) (Martin et al., 1991), which may translate into psychoactive side effects in humans (e.g., drowsiness, dizziness, ataxia, and fatigue).

A growing body of evidence indicates that in the treatment of chronic pain conditions, stimulation of the endocannabinoid system presents a promising approach that may prevent the occurrence of CNS side effects (Lomazzo et al., 2015). Several new strategies on how to preserve analgesic activity and avoid psychoactivity of cannabinoids have been proposed and tested in animals. They include inhibition of endocannabinoid uptake and metabolism in identified tissues where increased levels of endocannabinoids are desirable, administration of novel compounds that selectively target peripheral CB1 and CB2 receptors, positive allosteric modulation of cannabinoid CB1 receptor signaling, and modulation of non-CB1/non-CB2 receptors (TRPV1, GPR55, and PPARs) (Malek and Starowicz, 2016; Starowicz and Finn, 2017). In recent years, dual-acting compounds that provide FAAH inhibition (increased AEA and decreased arachidonic acid levels), TRPV1 antagonism (that prevents activation of the pro-nociceptive pathway by AEA), or COX-2 inhibition (that increases AEA and decreases prostaglandin levels), have offered the most promising results in chronic pain states in animals (Maione et al., 2007; Grim et al., 2014; Morera et al., 2016; Malek and Starowicz, 2016; Aiello et al., 2016; Starowicz and Finn, 2017). However, it is important to verify whether the efficacy of this multi-target strategy observed in rodent models of chronic pain and inflammation translates to humans and is not species-specific.

### Neuropathic Pain

Cannabinoids have been studied in various types of neuropathic pain in animals, including chronic nerve constriction traumatic nerve injury, trigeminal neuralgia, chemotherapy- and streptozotocin-induced neuropathy, etc.

Both CB1 and CB2 receptors have been found to be upregulated in nervous structures involved in pain processing in response to peripheral nerve damage (Lim et al., 2003; Zhang et al., 2003; Hsieh et al., 2011), and this may explain the beneficial effects of cannabinoid receptor agonists on neuropathic pain. It has been shown that increased CB2 expression is accompanied by the appearance of activated microglia (Zhang et al., 2003). Both microglial activation and neuropathic pain symptoms can be suppressed by CB2 agonists (Wilkerson et al., 2012). Consistent with this, CB2 knockout mice and transgenic mice overexpressing CB2 are characterized by enhanced and suppressed reactivity of microglia and neuropathic pain symptoms, respectively (Racz et al., 2008). TRPV1 expression is also increased in glutamatergic neurons of the medial prefrontal cortex in a model of spared nerve injury (SNI) in rats (Giordano et al., 2012).

In different neuropathic pain conditions, systemic administration of synthetic mixed cannabinoid CB1/CB2 agonists produces antinociceptive effects similar to those of THC (Herzberg et al., 1997; Pascual et al., 2005; Liang et al., 2007). The CB2 selective agonists given intrathecally or systemically are also effective in several animal models of neuropathic pain (Yamamoto et al., 2008; Kinsey et al., 2011), but their antinociceptive effects are without development of tolerance, physical withdrawal and other CNS side effects that accompany CB1 agonism (Deng et al., 2015).

When given early in the course of diabetes, CBD attenuates microgliosis in the ventral lumbar spinal cord of diabetic mice, as well as tactile allodynia and thermal hyperalgesia. However, if given later in the course of the disease, CBD has a little effect on pain-related behavior (Toth et al., 2010).

A controlled cannabis extract containing numerous cannabinoids and other non-cannabinoid fractions such as terpenes and flavonoids demonstrated greater antinociceptive efficacy than the single cannabinoid given alone, indicating synergistic antinociceptive interaction between cannabinoids and non-cannabinoids in a rat model of neuropathic pain (Comelli et al., 2008). The anti-hyperalgesic effect did not involve the cannabinoid receptors but was mediated by TRPV1 and thus it most probably belongs to CBD.

In animals with neuropathic pain, increased levels of endocannabinoids (AEA and 2-AG) have been detected in different regions of the spinal cord and brain stem (Mitrirattanakul et al., 2006; Petrosino et al., 2007). However, they appeared to be differentially regulated in different models of neuropathic pain, depending on the characteristic of the pain and the affected tissues (Starowicz and Przewlocka, 2012). Genetic or pharmacological inactivation of FAAH/MAGL resulting in the elevation of endocannabinoid (AEA/2-AG) levels in the spinal cord and brain stem (Lichtman et al., 2004; Schlosburg et al., 2009; Long et al., 2009; Adamson Barnes et al., 2016) show promise for suppressing both neuropathic and inflammatory pain. In general, the antinociceptive effect of endocannabinoids is sensitive to antagonists of CB1 and CB2 receptors, TRPV1 channels and PPARα antagonism, indicating that multiple targets could be involved in the mechanism of their action (Kinsey et al., 2010; Caprioli et al., 2012; Piomelli, 2014; Adamson Barnes et al., 2016). The reduction in the side effects that accompany CB1 agonism, such as motor incoordination, catalepsy, sedation and hypothermia, suggests that mainly TRPV1, but not a cannabinoid receptor-dependent mechanism, mediate the analgesic properties of exogenously and endogenously elevated levels of AEA in neuropathic pain. In a rat chronic constriction injury (CCI) model, depending on the dose of URB597 (FAAH inhibitor) used, lower or higher elevation of endogenous AEA levels and CB1- or TRPV1-mediated analgesia were achieved, respectively (Starowicz et al., 2012). It has been suggested that endocannabinoids can increase the excitability of nociceptive neurons by reducing synaptic release of inhibitory neurotransmitters via CB1 receptors on dorsal horn neurons (Pernía-Andrade et al., 2009), as well as by agonist activity on TRPV1 (Ross, 2003).

Monoacylglycerol lipase inhibitors demonstrated CB1-dependent behavioral effects, including analgesia, hypothermia and hypomotility (Long et al., 2009). In a mouse model of neuropathic pain both CB1 and CB2 were engaged in the anti-allodynic effects of FAAH inhibitors, while only CB1 was involved in the anti-allodynic effect of the MAGL inhibitor (Kinsey et al., 2010). Also, unlike FAAH inhibitors, the persistent blockade of MAGL activity leads to desensitization of brain CB1 receptors and loss of the analgesic phenotype (Chanda et al., 2010) and physical dependence (Schlosburg et al., 2009). However, a new highly selective MAGL inhibitor, KML29, exhibited antinociceptive activity without cannabimimetic side effects (Ignatowska-Jankowska et al., 2014).

In CCI in mice, JZL195, a dual inhibitor of FAAH and MAGL, demonstrated greater anti-allodynic efficacy than selective FAAH or MAGL inhibitors, and a greater therapeutic window (less motor incoordination, catalepsy and sedation) than WIN55212, a cannabinoid receptor agonist (Adamson Barnes et al., 2016).

Co-administration of sub-threshold doses of FAAH inhibitor, PF-3845 and the non-selective COX inhibitor, diclofenac sodium, produced enhanced antinociceptive effects in rodent models of both neuropathic (CCI) and inflammatory pain (intra-plantar carrageenan) (Grim et al., 2014). Combined FAAH inhibition/TRPV1 antagonism is also an attractive therapeutic strategy because FAAH inhibition only produced biphasic effects, with antinociception via CB1 at low levels of AEA, and when AEA levels were higher, pronociceptive effects via TRPV1 (Maione et al., 2007; Malek and Starowicz, 2016).

Cannabinoids may attenuate neuropathic pain by peripheral action via both CB1 and/or CB2 receptors (Fox et al., 2001; Elmes et al., 2004). The peripherally acting cannabinoid agonist AZ11713908 reduced mechanical allodynia with a similar efficacy to WIN55,212-2, an agonist that entered the CNS (Yu et al., 2010). In addition, URB937, a brain impermeant inhibitor of FAAH, elevated anandamide outside the brain and controlled neuropathic pain behavior without producing CNS side effects (Clapper et al., 2010).

After identification of allosteric binding site(s) on the CB1 GPCR (Price et al., 2005), several CB1-positive allosteric modulators have been developed and tested in animals. They attenuated both inflammatory and neuropathic pain behavior without producing the CB1-mediated side effects of orthosteric CB1 agonists but did not produce tolerance after repeated administration (Khurana et al., 2017; Slivicki et al., 2017).

### Inflammatory Pain

Different classes of cannabinoids (i.e., CB1 agonists, CB2 agonists, mixed CB1/CB2 agonists, endocannabinoids and endocannabinoid modulators) all suppressed pain behavior in various animal models of inflammatory pain (Clayton et al., 2002; Burgos et al., 2010; Starowicz and Finn, 2017). Since inflammatory pain is a characteristic of several chronic diseases, including cancer, arthritis, inflammatory bowel disease, sickle-cell disease, etc., cannabinoids appear to promise the lessening of severe pain in these diseases (Fichna et al., 2014; Abrams and Guzman, 2015; Turcotte et al., 2016; Vincent et al., 2016).

It is well known that CB2 receptor expression increases in microglia in response to inflammation and serves to regulate neuroimmune interactions and inflammatory hyperalgesia (Dhopeshwarkar and Mackie, 2014). However, the extent of CB2 expression in neurons is a subject of controversy (Atwood and Mackie, 2010; Atwood et al., 2012). It has been suggested that peripheral inflammation, unlike peripheral nerve injury, does not induce CB2 receptor expression in the spinal cord (Zhang et al., 2003). In contrast, Hsieh et al. (2011) demonstrated that the CB2 receptor gene is significantly upregulated in DRG and paws ipsilateral to inflammation induced by injection of complete Freund’s adjuvant (CFA).

Systemic or local peripheral injection of the CB2-selective agonist was reported to reduce nociceptive behavior and swelling in different animal models of inflammation (Quartilho et al., 2003; Elmes et al., 2005; Kinsey et al., 2011). In addition, the CB2-selective agonist did not produce hypothermia or motor deficit that are CB1-mediated side effects (Kinsey et al., 2011). Therefore, a CB2 receptor selective agonist is expected to have less psychomimetic side effects and lower abuse potential as compared to the available non-selective or CB1-selective cannabinoid agonists. In animal models of inflammatory disease, CB2 agonists slow the progression of diseases (Turcotte et al., 2016). In a murine model of rheumatoid arthritis, collagen-induced arthritis (CIA), CB2-selective agonists did not prevent the onset of arthritis, but did ameliorate established arthritis (Sumariwalla et al., 2004). JWH133, a selective CB2 agonist, inhibited *in vitro* production of cytokines in synoviocytes and *in vivo* reduced the arthritis score, inflammatory cell infiltration and bone destruction in CIA (Fukuda et al., 2014). Another CB2-selective agonist, HU-308, was shown to reduce swelling, synovial inflammation and joint destruction, in addition to lowering circulating antibodies against collagen I in CIA (Gui et al., 2015). Although approved in a range of preclinical models of pain, LY2828360, CB2 agonist, failed in a trial of patients with osteoarthritic knee pain (Pereira et al., 2013).

It was shown that formalin administration to the hind paw of rats induced AEA release into the periaqueductal gray matter (Walker et al., 1999). FAAH knockout mice and mice that express FAAH exclusively in nervous tissue, displayed anti-inflammatory and anti-hyperalgesic effects in both the carrageenan and CIA models, and the effects were prevented by administration of a CB2 but not a CB1 antagonist (Lichtman et al., 2004; Kinsey et al., 2011). FAAH inhibition may also reduce nociceptive behavior induced by lipopolysaccharide injection into the rat hind paw, and examination of the mechanism showed that both CB1 and CB2 were involved, but not TRPV1, PPARs, or opioid receptors (Booker et al., 2012). Oral administration of PF-04457845, a highly efficacious and selective FAAH inhibitor, produced potent antinociceptive effects in the CFA model of arthritis in rats, and it was shown that both CB1 and CB2 receptors were implicated in this effect (Ahn et al., 2011). In contrast to animal data, PF-04457845 failed to demonstrate efficacy in a randomized placebo and active-controlled clinical trial on pain in osteoarthritis of the knee (Ahn et al., 2011; Huggins et al., 2012). The possible explanations are development of tolerance to chronically elevated endocannabinoids or sensitization of TRPV1 receptors. A pronociceptive phenotype has been recently documented in FAAH knockout mice after administration of a challenge dose of TRPV1 agonist capsaicin (Carey et al., 2016). The increased nociceptive response was attenuated by antagonists of CB1 and TRPV1 receptors.

In a recent phase I trial, the FAAH inhibitor BIA-102474 caused death in one and severe neurological damage in five participants (Kaur et al., 2016; Moore, 2016). It has been suggested that specificity and non-selectivity of this molecule and several errors in the design of the study were responsible for its toxicity, and not targeting of FAAH *per se* (Huggins et al., 2012; Pawsey et al., 2016). More research is necessary to characterize both the efficacy and safety profiles of endocannabinoid-directed therapeutic strategies.

An increase in local endocannabinoid levels by inhibition with local peripheral administration of URB597 (an irreversible FAAH inhibitor) induced analgesia in a model of carrageenan-induced inflammation in rats that was inhibited by a PPARα antagonist but not by a CB1 receptor antagonist (Sagar et al., 2008). However, local administration of URB597 into osteoarthritic knee joints reduced pain via CB1 receptors [monosodium iodoacetate (MIA)-induced osteoarthritis in rats and the model of spontaneous osteoarthritis in Dunkin-Hartley guinea pigs] (Schuelert et al., 2011). A peripherally restricted FAAH inhibitor, URB937, also reduced inflammatory pain in rodents *via* CB1 receptors (Clapper et al., 2010).

It was shown that inhibition of fatty acid binding proteins (FABPs) reduced inflammatory pain in mice. This effect was associated with an upregulation of AEA and the effect was inhibited by antagonists of CB1 or PPARα receptors (Kaczocha et al., 2014).

Recent animal findings suggest that cannabinoids may have beneficial effect on affective-emotional and cognitive aspect of chronic pain (La Porta et al., 2015; Neugebauer, 2015; Kiritoshi et al., 2016). In mice with MIA-induced arthritis, selective agonists of both CB1 and CB2 receptors ameliorated the nociceptive and affective manifestations of osteoarthritis, while a CB1-selective agonist improved the memory impairment associated with arthritis (La Porta et al., 2015; Woodhams et al., 2017). This is in agreement with human studies of cannabinoids that indicate a significant improvement in secondary outcome measures, such as sleep and mood (Lynch and Ware, 2015).

The combined FAAH/COX inhibitor ARN2508 demonstrated efficacy against intestinal inflammation and was without gastrointestinal side effects (Sasso et al., 2015) because AEA, which is similar to prostanoids, has protective actions on the gastrointestinal mucosa.

### Cancer Pain

Experiments with animal models of cancer pain support the use of cannabinoids in the treatment of cancer pain in humans. Systemic administration of non-selective, CB1 selective or CB2 selective agonist significantly attenuated mechanical allodynia in a mouse model which was produced by inoculating human oral cancer cell lines HSC3 into the hind-paw of mice (Guerrero et al., 2008). A mechanical hyperalgesia associated with decreased anandamide levels were found in plantar paw skin ipsilateral to tumor induced by injection of fibrosarcoma cells into the calcaneum of mice. The paw withdrawal frequency was reduced after local injection of anandamide (Khasabova et al., 2008). Also, one study reported that the efficacy of synthetic CB1- and CB2-receptor agonists was comparable with the efficacy of morphine in a murine model of tumor pain (Khasabova et al., 2011). An important finding is that cannabinoids are effective against neuropathic pain induced by exposure of animals to anticancer chemotherapeutics (vincristine, cisplatin, paclitaxel) (Rahn et al., 2007; Khasabova et al., 2012; Ward et al., 2014).

## Clinical Trials of Cannabis/Cannabinoids in Chronic Pain

Pain relief is the most commonly cited reason for the medical use of cannabis. In 2011, 94% of the registrants on the Medical Marijuana Use Registry in Colorado (United States) were using medical marijuana for chronic pain (Kondrad and Reid, 2013). However, cannabis is not the first drug of choice that a patient takes to relieve pain. As with many other analgesics, cannabinoids do not seem to be equally effective in the treatment of all pain conditions in humans. This is most probably due to the different mechanisms of pain (e.g., acute vs. chronic, or chronic non-cancer vs. chronic cancer pain) (Romero-Sandoval et al., 2017). Clinical studies have shown that cannabinoids are not effective against acute pain (Buggy et al., 2003; Beaulieu, 2006; Holdcroft et al., 2006; Kraft et al., 2008). Clinical data also indicate that cannabinoids may only modestly reduce chronic pain, like all presently available drugs for the treatment of chronic pain in humans (Romero-Sandoval et al., 2017).

### Efficacy of Cannabis/Cannabinoids in the Treatment of Chronic Pain

Until recently, there was no consensus about the role of cannabinoids for the treatment of chronic pain. Several years ago, the European Federation of Neurological Societies recommended cannabinoids (THC, oromucosal sprays 2.7 mg delta-9-tetrahydrocannabinol/2.5 mg cannabidiol) as the second or third line of treatment of central pain in MS (Attal et al., 2010). More recently, the Canadian Pain Society supported their use as the third-line option for the treatment of neuropathic pain, after anti-convulsives, anti-depressants, and opioids (Moulin et al., 2014). In addition, Health Canada provided preliminary guidelines for prescribing smoked cannabis in the treatment of chronic non-cancer pain (Kahan et al., 2014). At the same time, the Special Interest Group on neuropathic pain of the International Association for the Study of Pain provided “a weak recommendation against the use of cannabinoids in neuropathic pain, mainly because of negative results, potential misuse, abuse, diversion and long-term mental health risks particularly in susceptible” (Finnerup et al., 2015).

There is a growing body of evidence to support the use of medicinal cannabis in the treatment of chronic pain. At present, there is a scientific consensus on the medicinal effects of cannabis for the treatment of chronic pain that is based on scientific evidence. The National Academy of Sciences, Engineering and Medicine (NASEM, 2017) has evaluated more than 10,000 scientific abstracts and established that there is “conclusive or substantial evidence” for the use of cannabis in treating chronic pain in adults. Also, there is “moderate evidence” that cannabinoids, in particular nabiximols, are effective in improving short-term sleep outcomes in patients with chronic pain (NASEM, 2017). The expert report NASEM supports more research to determine dose–response effects, routes of administration, side effects and risk-benefit ratio of cannabis/cannabinoid use with precision and make possible evidence based policy measure implementation. At the same time, the PDQ Integrative Alternative and Complementary Therapies Editorial Board (2018) states that pain relief is one of the potential benefits of cannabis/cannabinoids for people living with cancer (in addition to its anti-emetic effects, appetite stimulation, and improved sleep).

#### Chronic Non-cancer Pain

Lynch and Campbell (2011) and Lynch and Ware (2015) performed two systematic reviews of cannabis/cannabinoid use in chronic non-cancer pain (neuropathic pain, fibromyalgia, rheumatoid arthritis and mixed chronic pain) involving 18 randomized controlled trials published between 2003 and 2010, and 11 studies published between 2011 and 2014, respectively. All 29 trials included about 2000 participants and their duration was up to several weeks. Twenty-two of 29 trials demonstrated a significant analgesic effect and several also reported improvements in secondary outcomes (sleep, spasticity).

Whiting et al. (2015) performed a systematic review of the benefits and adverse events of orally administered cannabinoids and inhaled cannabis for a variety of indications (chronic pain was assessed in 28 studies, there were 2454 participants, the follow-up period lasted up to 15 weeks), and provided moderate-quality evidence to support the use of cannabinoids for the treatment of chronic pain.

The Canadian Agency for Drugs and Technologies in Health (2016) recently analyzed five systematic reviews (including two with meta-analyses) of nabiximols (THC:CBD buccal spray) for the treatment of chronic non-cancer or neuropathic pain (Lynch and Campbell, 2011; Lynch and Ware, 2015; Jawahar et al., 2013; Boychuk et al., 2015; Whiting et al., 2015). The length of the follow-up across the studies was from 1 to 15 weeks. In this review, there are inconsistencies with regard to both the effectiveness and safety of nabiximols. The authors concluded that treatment with nabiximols in the short term may be associated with pain relief and good tolerability when compared with placebo therapy, but there is still insufficient evidence to support its use in the management of chronic neuropathic and non-cancer pain.

##### Neuropathic pain

###### Cannabis

The meta-analysis of individual patient data from 5 randomized trials (178 participants) presents evidence that inhaled cannabis may provide short-term reductions (>30% reduction in pain scores) in chronic neuropathic pain (diabetes, HIV, trauma) for 1 in 5–6 patients (Andreae et al., 2015). In these trials, the THC content ranged from 3.5 to 9.4%. A dose-related effect of cannabis was found, with higher THC contents producing more pronounced pain relief. In one study, pain relief was not dose-dependent and was achieved with a low concentration of cannabis THC [1.29% (vaporized)] (Wilsey et al., 2013). The follow-up periods ranged from days to weeks. Consistent with the results of this meta-analysis, a more recent, small, randomized, double-blind, placebo-controlled crossover clinical study demonstrated that vaporized cannabis (1–7% THC) in a dose-dependent manner reduced spontaneous and evoked pain in patients (16 subjects) suffering from painful diabetic neuropathy (Wallace et al., 2015). The analgesic effect was achieved at THC concentrations as low as 1–4%. In a more recent randomized, placebo-controlled, double-blind crossover study (38–41 participants *per* group), Wilsey et al. (2016) reported that low THC concentrations (2.9–6.7%) of vaporized cannabis effectively reduced chronic neuropathic pain after spinal cord injury or disease. It was found that higher plasma levels of THC and/or the THC metabolite significantly correlated with improvements in clinical symptoms of pain (Wilsey et al., 2016).

###### Oral cannabinoids

No recommendations regarding cannabinoid treatment of non-spastic and non-trigeminal neuralgic pain in adult patients with MS were reported in the systemic review of Jawahar et al. (2013). Results of another systematic review that analyzed the effectiveness of cannabis extracts and cannabinoids in the treatment of chronic non-cancer neuropathic pain suggested that cannabis-based medicinal extracts may provide pain relief in conditions that are refractory to other treatments (Boychuk et al., 2015). It was pointed out that further studies are required to estimate the influence of the duration of the treatment.

A recently published systematic review (Meng et al., 2017) considered 11 randomized controlled studies involving a total of 1219 participants in which oral cannabinoids (dronabinol, nabilone, and nabiximols) were compared with standard pharmacological and/or non-pharmacological treatments or placebo in patients with neuropathic pain (including MS). This study shows that oral cannabinoids are modestly effective in reducing chronic neuropathic pain and that for this effect a minimum of 2 weeks of treatment is required. The study also showed improvements in the quality of life, sleep and increased patient satisfaction. However, the quality of the evidence is moderate and the strength of recommendation for analgesic efficacy of selective cannabinoids in this clinical setting is weak. Of the different cannabinoids used, nabiximols and dronabinol but not nabilone demonstrated an analgesic advantage.

The authors of the most recent Cochrane Review on the efficacy, tolerability and safety of cannabis-based medicines (CBM; botanical, plant-derived, and synthetic) compared to placebo or conventional drugs for neuropathic pain in adults (16 randomized, double-blind controlled trials with 1750 participants) concluded that the potential benefits of CBM in neuropathic pain might be outweighed by their potential harms (Mücke et al., 2018). All CBMs were superior to placebo in reducing pain intensity, sleep problems and psychological distress (very low- to moderate-quality evidence). Between these two groups, no differences were found in improvements to health-related quality of life and discontinuation of the medication because of its ineffectiveness. There was no difference between CBM and placebo in the frequency of serious adverse events (low-quality evidence). Adverse events were reported by 80.2% of participants in the CBM group and 65.6% of participants in the placebo group (RD 0.19, 95% CI 0.12–0.27; *P*-value < 0.0001; *I*^2^ = 64%). CBM may increase nervous-system adverse events compared with placebo [61% vs. 29%; RD 0.38 (95% CI 0.18–0.58); number-needed-to-harm (NNTH) 3 (95% CI 2–6); low-quality evidence], as well as psychiatric disorders (17% vs. 5%: RD 0.10 (95% CI 0.06–0.15); NNTH 10 (95% CI 7–16); low-quality evidence]. Some of the adverse events (e.g., somnolence, sedation, confusion, and psychosis) may limit the clinical usefulness of CBM.

##### Rheumatic pain

Four randomized controlled trials with 159 patients with fibromyalgia, osteoarthritis, chronic back pain and rheumatoid arthritis treated with cannabinoids (nabilone, nabiximols, and FAAH inhibitor) or placebo or an active control (amitriptyline), were included in a systemic review (Fitzcharles et al., 2016). The results were not consistent and did not reveal whether the cannabinoids were superior to the controls (placebo and amitriptyline). The authors concluded that there is insufficient evidence for the recommendation for cannabinoid use for pain management in patients with rheumatic diseases. Smoked cannabis has not been tested for pain relief in patients suffering from rheumatoid pain (Ko et al., 2016).

##### Chronic abdominal pain

In a randomized, double-blind, placebo-controlled parallel-design phase 2 study (65 participants), no difference between a THC tablet and a placebo tablet in reducing pain measures in patients with chronic abdominal pain due to surgery or chronic pancreatitis was found (de Vries et al., 2017).

#### Chronic Cancer Pain

Cancer pain is a chronic pain, often complex, consisting of nociceptive, inflammatory and neuropathic components. Severe and persistent cancer pain is often refractory to treatment with opioid analgesics (Abrams and Guzman, 2015).

Nabiximols has been considerably studied in patients with cancer pain. It has been conditionally approved in Canada and some European countries for the treatment of cancer-related pain. Currently, it is in phase 3 trials for cancer pain. A multicenter, double-blind, randomized, placebo-controlled study (177 patients) demonstrated that nabiximols (2.7 mg THC + 2.5 mg CBD) given for 2 weeks is superior to a placebo for pain relief in advanced cancer patients whose pain was not fully relieved by strong opioids (Johnson et al., 2010). A randomized, placebo-controlled, graded-dose trial with advanced cancer patients (88–91 per group) whose pain was not fully relieved by strong opioids, demonstrated significantly better pain relief and sleep with THC:CBD oromucosal spray following 35 days of treatment with lower doses (1–4 and 6–10 sprays/day), compared with placebo (Portenoy et al., 2012). In an open-label extension study of 43 patients with long-term use of the THC:CBD oromucosal spray there was no need for increasing the dose of the spray or the dose of other analgesics (Johnson et al., 2013). However, results of more recent studies differ from previous ones and are not promising for the use of nabiximols in the treatment of cancer pain. Namely, two studies (multicenter, randomized, double-blind, placebo-controlled, and parallel-group) conducted by GW Pharmaceuticals, the manufacturer of nabiximols, suggested that the effects of nabiximols in patients with cancer pain resistant to opioid analgesics were not different from placebo (Fallon et al., 2017). In fact, it was shown that nabiximols is superior to placebo in a patient sub-population studied in the United States, but not in sub-populations studied outside of United States, and this finding warrants further examination.

At present, there is insufficient evidence to support the approval of dronabinol and nabilone for the treatment of any type of pain, including cancer pain. In an observational study of patients with advanced cancer, nabilone improved management of pain, nausea, anxiety and distress when compared with untreated patients. Nabilone was also associated with a decreased use of opioids and other pain killers, as well as dexamethasone, metoclopramide, and ondansetron (Maida et al., 2008). Two studies examined the effects of dronabinol on cancer pain. In the first, randomized, double-blind, placebo-controlled, dose-ranging study involving ten patients, significant pain relief was obtained with 15- and 20-mg doses; however, a 20-mg dose induced somnolence (Noyes et al., 1975b). In a follow-up, single-dose study involving 36 patients, doses of 10 and 20 mg of dronabinol produced analgesic effects that were equivalent to doses of 60 and 120 mg of codeine, respectively (Noyes et al., 1975a). However, higher doses of dronabinol were found to be more sedating than codeine. It can be concluded that the effectiveness of cannabinoids in the treatment of chronic cancer pain is questionable. However, whether cannabinoids show some other improvements in cancer patients (sleep, quality of life) remains to be explored. More research is required to establish the role of cannabinoids in the treatment of cancer pain.

There are some case studies, but no published controlled clinical trials, on the use of inhaled cannabis for the treatment of pain in patients with cancer. Also, inhaled cannabis could be effective against chemotherapy-induced neuropathic pain in patients with cancer (Wilsey et al., 2013; Wilsey et al., 2016).

### Tolerability and Safety of Cannabis/Cannabinoids in the Treatment of Chronic Pain

#### Short-Term Tolerability and Safety

Findings from available short-term clinical studies suggest that the safety profile of the short-term use (days to weeks) of cannabis/cannabinoids for pain treatment is acceptable. Their short-term use was associated with an increased risk of adverse events, but they were mostly mild and well tolerated (Wang et al., 2008; Lynch and Campbell, 2011; Andreae et al., 2015; Lynch and Ware, 2015; Whiting et al., 2015; Meng et al., 2017). The psychoactive effects of inhaled cannabis were dose-dependent, rare and mild in intensity (Andreae et al., 2015). The treatment with oral cannabinoids was associated with limited tolerability. They produce more cannabinoid-related side effects than placebo, but the side effects are mild to moderate and short-lived (Meng et al., 2017).

One systematic review of safety studies (23 RCTs and 8 observational studies) of medical cannabis and cannabinoids found that short-term use appeared to increase the risk of non-serious adverse events and that they represent 96.6% of all reported adverse events (Wang et al., 2008). Usually no difference in the incidence rate of serious adverse events was found between the group of patients assigned medical cannabis/cannabinoids and the control group. Psychiatric adverse effects are the most common reason for withdrawal of the treatment. The most commonly reported adverse effect was dizziness (15.5%), followed by drowsiness, faintness, fatigue, headache, problems with memory and concentration, the ability to think and make decisions, sensory changes, including lack of balance and slower reaction times (increased motor vehicle accidents), nausea, dry mouth, tachycardia, hypertension, conjunctival injection, muscle relaxation, etc. (Wang et al., 2008; Belendiuk et al., 2015). Tolerance to these adverse effects develops soon after the beginning of treatment. Cannabis/cannabinoids can cause mood changes or a feeling of euphoria, dysphoria, anxiety and even hallucinations and paranoia. They can also worsen depression, mania or other mental illnesses. Due to lack of cannabinoid receptors in the brainstem areas controlling respiration, lethal overdoses from cannabis do not occur.

#### Long-Term Tolerability and Safety

As cannabis/cannabinoids are intended for treating chronic pain conditions, their long-term tolerability and safety has to be precisely determined, as do the potential health effects of recreational cannabis use (Mattick, 2016). The brain develops a tolerance to cannabinoids, and long-term studies with cannabinoids need to answer the question whether pain can be constantly controlled with these drugs, or whether tolerance and a hyperalgesic response can occur. However, at present there are few well-designed clinical trials and observational studies for long-term medicinal cannabis use that have examined tolerability and safety (mostly in MS patients and in use of oral cannabinoids).

One controlled (open-label) study has evaluated the safety and tolerability of cannabis (a standardized botanical cannabis product that contains 12.5% tetrahydrocannabinol) used for 1 year in 215 patients (from seven clinics across Canada) with chronic non-cancer pain (Ware et al., 2015). There was a higher rate of adverse events (mostly mild to moderate with respect to the nervous system and psychiatric disorders) among cannabis users when compared to controls, but not for serious adverse events at an average dose of 2.5 g botanical cannabis per day. The conclusion of the authors of this study is that cannabis is tolerated well and relatively safe when used long-term. The beneficial effect persists over time, indicating that cannabis use for over 1 year does not induce analgesic tolerance.

The effectiveness and long-term safety of cannabinoid capsules (2.5 mg dronabinol vs. cannabis extract containing 2.5 mg THC, 1.25 mg CBD vs. placebo) in MS (630 patient) was studied in a 1-year randomized, double-blind, placebo-controlled trial follow-up of a randomized parent study (Zajicek et al., 2005). The number of patients who withdrew due to side effects was similar between groups. Also, serious side effects were similar in the placebo and active groups and were related to the medical condition. Generally, there were no safety concerns reported in this study.

The safety and tolerability of nabiximols long-term use in different conditions (cancer pain, spasticity and neuropathic pain in MS patients) has been studied in a series of trials of up to 2 years duration (Wade et al., 2006; Rog et al., 2007; Johnson et al., 2013; Serpell et al., 2013). All were uncontrolled, open-label extension trials. Adverse events and serious adverse events were cannabinoid-related with no safety concerns reported. Also, there was no evidence for a loss of effect in the relief of pain with long-term use.

Taking into account all long-term safety studies, cannabis appears to be better tolerated than oral cannabinoids (Romero-Sandoval et al., 2017). This interpretation is based on a single study with cannabis (Ware et al., 2015) and should therefore be taken with caution.

Long-term adverse effects of medical cannabis are difficult to evaluate. They mainly come from studies with recreational cannabis use (Mattick, 2016). However, there are many differences between medical cannabis and recreational cannabis users as regards the amounts used, the existence of comorbidities, the mode of drug delivery (Wang et al., 2008), etc. Thus, the adverse effects of recreational cannabis use cannot be directly extrapolated to medical cannabis use. The safety of medical and recreational cannabis should be evaluated separately. There is evidence that long-term cannabis use is associated with an increased risk of addiction, cognitive impairment, altered brain development and an increased risk of mental disorders (anxiety, depression, and psychotic illness) with adolescent use, and adverse physical health effects such as cardiovascular disease, chronic obstructive pulmonary disease and lung cancer (Volkow et al., 2014; Mattick, 2016). It is well established and documented that CBD may lower the risk for developing psychotic illness that is related to cannabis use (Iseger and Bossong, 2015).

Cannabis-use disorders (CUD) are defined in the Diagnostic and Statistical Manual of Mental Disorders (Hasin et al., 2013) and in the International Statistical Classification of Diseases and Related Health Problems (ICD-11). It was estimated that 9% of those who use cannabis develop CUD (Budney et al., 2007). Risk factors (e.g., cannabis use at an earlier age, frequent use, combined use of abused drugs) for the progression of cannabis use to problem cannabis use (CUD, dependence, and abuse) (NASEM, 2017; Hasin, 2018) are more common among recreative than among medical cannabis users. CUD are associated with psychiatric comorbidities. About one half of patients treated for CUD develop withdrawal symptoms such as dysphoria (anxiety, irritability, depression, and restlessness), insomnia, hot flashes and rarely gastrointestinal symptoms. These symptoms are mild when compared with withdrawal symptoms associated with opioid use. Most of the symptoms appear during the 1st week of cannabis withdrawal and resolve after a few weeks (Gordon et al., 2013; Volkow et al., 2014).

A number of studies have yielded conflicting evidence regarding the risks of various cancers associated with cannabis smoking (Health Canada, 2013). Recently, NASEM (2017) has stated, with a moderate level of evidence, that there is no statistical association between cannabis smoking and lung cancer incidence.

Before grant approval, drug agencies need to be sure that the benefits of medicine outweigh the risks. As the benefits and risks of medical cannabis have not been thoroughly examined, individual products containing cannabinoids have not been approved for the treatment of pain (Ko et al., 2016). Nonetheless, a number of chronic-pain patients use cannabis/cannabinoids for pain relief. Some replaced partially or completely the use of opioids with cannabis/cannabinoids (Boehnke et al., 2016; Lucas and Walsh, 2017; Lucas, 2017; Piper et al., 2017), and others continued to use prescription opioids. Observational studies have found that state legalization of cannabis is associated with a decrease in opioid addiction and opioid-related over-dose deaths (Hayes and Brown, 2014; Powell et al., 2018). Previous studies suggested that the analgesic effects of cannabis are comparable to those of traditional pain medications (Wilsey et al., 2013). However, data on the comparative efficacy and safety of cannabis/cannabinoids versus existing pain treatments, including opioids, are missing. Also, more studies are needed on potentially beneficial or problematic combinations of cannabis/cannabinoids and available analgesics. Further research is expected to provide an answer to the question whether cannabis/cannabinoids can be an effective and safe substitute for opioid therapy in the treatment of chronic pain (Nielsen et al., 2017). New high-quality, long-term exposure trials are required to determine the efficacy and safety of long-term use of medicinal cannabis in the treatment of pain (Hill et al., 2017; Piomelli et al., 2017; Romero-Sandoval et al., 2017). The design of trials should be improved to ensure that they are blinded, placebo-controlled with active comparator, with consistency of pain diagnosis, long-enough duration of treatment, evaluation of the dose-response, homogeneity of the patient population and inclusion of quality of life as an outcome measure (Ko et al., 2016; NASEM, 2017; Piomelli et al., 2017).

Current research evidence supports the use of medical cannabis in the management of chronic pain in adults (NASEM, 2017). As its use in the treatment of chronic pain increases, additional research to support or refute the current evidence base is crucial to provide answers to questions concerning the risk-benefit ratio for medical cannabis use in pain treatment. The implementation of monitoring programs is mandatory and provides an opportunity to accumulate data on the safety and effectiveness of long-term use of medical cannabis in the real world (Hill et al., 2017; Romero-Sandoval et al., 2017). This is important for evidence-based policy making and implementation (Nosyk and Wood, 2012).

## Summary

The key findings are summarized below:

Cannabinoids and cannabis are old drugs but now they are a promising new therapeutic strategy for pain treatment.

Cannabinoids (plant-derived, synthetic) themselves or endocannabinoid-directed therapeutic strategies have been shown to be effective in different animal models of pain (acute nociceptive, neuropathic, inflammatory). However, medical cannabis is not equally effective against all types of pain in humans.

A recent meta-analysis of clinical trials of medical cannabis for chronic pain found substantial evidence encouraging its use in pharmacotherapy of chronic pain. Also, it was shown that medical cannabis may only moderately reduce chronic pain, similar all other currently available analgesic drugs. However, controlled comparative studies on the efficacy and safety of cannabis/cannabinoids and other analgesics, including opioids, are missing.

Inhaled (smoked or vaporized) cannabis is constantly effective in reducing neuropathic pain and this effect is dose-related and can be achieved with a concentration of cannabis THC lower than 10%. Compared to oral cannabinoids, the effect of inhaled cannabis is more rapid, predictable and can be titrated. Compared to inhaled cannabis, the effectiveness of oral cannabinoids in reducing the sensory component of neuropathic pain seems to be less convincing and oral cannabinoids in general may be less tolerable. However, data suggest that they may improve secondary measures such as sleep, quality of life and patient satisfaction.

There are no controlled clinical trials on the use of inhaled cannabis for the treatment of cancer or rheumatic (osteoarthritis, rheumatoid arthritis, and fibromyalgia) pain.

Whether oral cannabinoids reduce the intensity of chronic cancer pain is not completely clear. Recent long-term studies of nabiximols are not encouraging.

Sparse literature data show that oral cannabinoids have inadequate efficacy in rheumatological pain conditions. Also, oral cannabinoids do not reduce acute postoperative or chronic abdominal pain.

In general, the efficacy of medical cannabis in pain treatment is not completely clear due to several limitations. Clinical trials are scarce and most were of short duration, with relatively small sample sizes, heterogeneous patient populations, different types of cannabinoids, a range of dosages, variability in the assessment of domains of pain (sensory, affective) and modest effect sizes. Therefore, further larger studies examining specific cannabinoids and strains of cannabis, using improved and objective pain measurements, appropriate dosages and duration of treatment in homogeneous patient populations need to be carried out.

The current review of evidence from clinical trials of medicinal cannabis suggests that the adverse effects of its short-term use are modest, most of them are not serious and are self-limiting.

Long-term safety assessment of medicinal cannabis is based on scant clinical trials, so the evidence is limited, and the safety interpretation should be taken cautiously. More research is needed to evaluate the adverse effects of long-term use of medical cannabis.

In view of the limited effect size and the low but not unimportant risk of serious, adverse events, a more precise determination of the risk-to-benefit ratio for medicinal cannabis in pain treatment is needed to help establishing evidence-based policy implementation.

Current evidence supports the use of medical cannabis in the treatment of chronic pain in adults. Monitoring and follow-up of patients is obligatory.

## Author Contributions

SV conceived and wrote manuscript. DS participated in literature search. All authors revised the manuscript and approved the final manuscript for submission.

## Conflict of Interest Statement

The authors declare that the research was conducted in the absence of any commercial or financial relationships that could be construed as a potential conflict of interest.
